# Anticrystal Engineering of Ketoprofen and Ester Local Anesthetics: Ionic Liquids or Deep Eutectic Mixtures?

**DOI:** 10.3390/pharmaceutics12040368

**Published:** 2020-04-17

**Authors:** Anita Umerska, Klaudia Bialek, Julija Zotova, Marcin Skotnicki, Lidia Tajber

**Affiliations:** 1 School of Pharmacy and Pharmaceutical Sciences, Trinity College Dublin, College Green, Dublin 2, Ireland; umerskaa@tcd.ie (A.U.); bialekk@tcd.ie (K.B.); zotovaj@tcd.ie (J.Z.); marcskot@ump.edu.pl (M.S.); 2 Department of Pharmaceutical Technology, Poznań University of Medical Sciences, 60-780 Poznań, Poland

**Keywords:** ionic liquids, deep eutectic mixtures, ketoprofen, non-steroidal anti-inflammatory drugs (NSAIDs), local anesthetics, procaine, benzocaine, tetracaine, anticrystal engineering, mechanochemistry

## Abstract

Ionic liquids (ILs) and deep eutectic mixtures (DEMs) are potential solutions to the problems of low solubility, polymorphism, and low bioavailability of drugs. The aim of this work was to develop and investigate ketoprofen (KET)-based ILs/DEMs containing an ester local anesthetic (LA): benzocaine (BEN), procaine (PRO) and tetracaine (TET) as the second component. ILs/DEMs were prepared via a mechanosynthetic process that involved the mixing of KET with an LA in a range of molar ratios and applying a thermal treatment. After heating above the melting point and quench cooling, the formation of supercooled liquids with Tgs that were dependent on the composition was observed for all KET-LA mixtures with exception of that containing 95 mol% of BEN. The KET-LA mixtures containing either ≥ 60 mol% BEN or 95 mol% of TET showed crystallization to BEN and TET, respectively, during either cooling or second heating. KET decreased the crystallization tendency of BEN and TET and increased their glass-forming ability. The KET-PRO systems showed good glass-forming ability and did not crystallize either during the cooling or during the second heating cycle irrespective of the composition. Infrared spectroscopy and molecular modeling indicated that KET and LAs formed DEMs, but in the KET-PRO systems small quantities of carboxylate anions were present.

## 1. Introduction

The pharmaceutical industry relies on solid, predominantly crystalline, forms of active pharmaceutical ingredients (APIs) [1]. Crystalline APIs, including multicomponent systems such as salts and co-crystals, exhibit polymorphism that could negatively influence the solubility and bioavailability of the API [2]. Ionic liquids (ILs) have recently been identified as a promising approach to overcome the above-mentioned problems [3,4,5]. The IL form can improve the API performance by providing controlled solubility and drug delivery [6,7]. An IL is a multicomponent, one-phase system composed of ionized species with either a melting point (Mp) or a glass transition temperature (Tg) below 100 °C [6]. However, it has been shown that not only the proton transfer, but also strong hydrogen bond formation can be the driving force for the liquefaction of solid APIs, leading to the formation of deep eutectic mixtures (DEMs) [4,8,9]. For instance, it has been demonstrated that lidocaine ibuprofenate, generally considered as an IL [10], is in fact a DEM due to the low degree of ionization/proton transfer [2]. APIs are transformed into ILs by combining them with appropriate counterions. These counterions should ideally be monovalent, with a minimal number of potential H-bonds between molecules, asymmetric, with soft electron density, bulky with voluminous side chains causing steric inhibition crystallization among the salt components [6]. The primary aim of the counterion is to lower the Mp of the salt, yielding a low-melting point phase. However, these counterions can modify the properties such as solubility, lipophilicity, partition coefficient, thereby affecting the API transport via biological membranes, its absorption and/or other pharmacokinetic parameters [1,2,3,4,10].

Interestingly, some APIs satisfy the above-mentioned IL counterion requirements [6]. The dual functional ILs (i.e., ILs composed of two APIs) offer the advantage of lowering of the Mp of API, in addition to providing a second biological function [2,3,4]. The potential clinical benefits and the increasing research and development cost of each new molecular entity have attracted interest in developing drug-drug combinations [11,12]. Hence, the possibility of assembly of drug combination in the form of ILs or DEMs, whereby two API molecules are associated together via reversible intermolecular interactions, is a very attractive approach, especially for pharmaceutical combination products. For instance, non-steroidal anti-inflammatory drugs (NSAIDs) and local anesthetics (LAs) could create a successful dual drug combination because both are widely used in the treatment of acute and chronic pain, as non-opioid strategies for pain management [13]. NSAIDs are generally acids, whereas LAs are bases, hence there is a potential for a proton exchange. There have been attempts to synthesize NSAID-LA ILs, with ibuprofen being the most commonly used NSAID and lidocaine, the most commonly used LA [2,10,14]. Ketoprofen (KET) is another very interesting and promising NSAID candidate to form ILs and information on KET-based ILs is scarce, with only tetrabutylphosphonium [7] and choline [15] used as the counterions. To the best of authors’ knowledge, there is no reports describing KET ILs or DEMs with LAs. Among LAs from ester group, there are two interesting APIs with low melting point that meet the IL counterion requirements: tetracaine (TET) and procaine (PRO). Another important compound from the ester LAs group, benzocaine (BEN), has a high crystallization tendency [16] and the presence only a weak base group (aromatic amine), thus providing a challenge for the IL formation.

The great majority of studies conducted thus far have focused on equimolar mixtures of the NSAID and LA [5,10,17], whereas only three NSAID-LA examples of complete phase diagrams, i.e., lidocaine-ibuprofen [2], lidocaine-indomethacin [18], and lidocaine-naproxen [19] have been described. However, due to the crucial importance of non-ionic interactions in DEMs, it is important to consider a wide variety of component ratios in addition to the equimolar mixture.

ILs are usually synthesized by metathesis reactions, which involve dissolving of the salts of the acid and the base components of ILs in a suitable solvent such as water, methanol, ethanol, within which the IL readily forms [6]. However, metathesis has considerable disadvantages such as the need for product purification, removal of counterions such as sodium, chloride, use of organic solvents and compromised product yield. Alternatively, the solvent-free process that involves co-melting of free forms of the acid and base components was used by Cherukuvada and Nangia [20] to obtain the ILs of ethambutol such as ethambutol adipate. 

The aim of this work was to develop and characterize KET-ester LAs ILs/DEMs at various mixing ratios and construct the phase diagrams to inform selection of the compositions with pharmaceutical relevance. Heating was employed to obtain ILs/DEMs via mechanosynthesis and to understand the thermal aspects of KET-LA mixing. The glass-forming ability and/or crystallization tendency of the studied mixtures was also investigated, and solid-state phase identification conducted. Infrared spectroscopy was the method employed to investigate the nature of the interaction between the components (i.e., H-bonding versus proton transfer) and to ultimately determine whether they form a DEM or an IL. To support the experimental efforts, molecular modelling was employed to understand intermolecular interactions in the KET-LA mixtures. 

## 2. Materials and Methods 

### 2.1. Materials

Ketoprofen (KET) was purchased from Fluorochem Ltd., (Hadfield, UK). Benzocaine (BEN), procaine (PRO) and tetracaine (TET) were purchased from TCI (Tokyo Chemical Industry UK Ltd., Oxford, UK). All chemicals were used as supplied and had purity of at least 98%.

### 2.2. Sample Preparation

The binary mixtures were prepared via mixing of an acid (KET) with a base (an LA). A quantity of 200 mg of each powder mixtures containing KET and each of the LA bases was prepared at a range of KET/LA mol% between 5 and 95. The constituents were accurately weighed using an MT5 Mettler Toledo microbalance (Greifensee, Switzerland) and ground in an agate mortar using an agate pestle until a homogenous powder mixture was obtained. 

### 2.3. Thermogravimetric Analysis (TGA)

TGA was performed to evaluate the thermal stability of samples to make sure that they do not decompose during heating. TGA was carried out on the powders using a Mettler Toledo TG50 measuring module coupled to a Mettler Toledo MT5 balance [21]. Approximately 8−10 mg samples were analyzed (*n* = 3) in open aluminum pans, using nitrogen as the purge gas with a flow rate of 40 mL/min. Samples were heated from 25 to 250 °C at a rate of 10 °C/min. Mettler Toledo STAR^e^ software (version 6.10) was used to determine the mass loss based on the slope of TGA trace. Samples were considered as stable at temperatures at which the mass loss was smaller than 5% of the initial sample weight.

### 2.4. Differential Scanning Calorimetry (DSC)

DSC was carried out using a Perkin Elmer Diamond DSC unit (Waltham, MA, USA) with a ULSP B.V. 130 cooling system (Ede, Netherlands). Nitrogen with a flow rate of 40 mL/min was employed as a purge gas that was controlled with a PerkinElmer Thermal Analysis Gas Station. The instrument was calibrated for temperature and heat flow using an indium calibration reference material (99.999%, transition point 156.60 °C) supplied by Perkin Elmer. Approximately 3–5 mg of accurately weighed powder (MT5 Mettler Toledo microbalance) were analyzed in 18 µL aluminum pans with covers. The samples were held at either 25 °C (KET-BEN) or 0 °C (KET-PRO and KET-TET) for 1 min in the DSC unit, then heated at a rate of 10 °C/min (=first heating), cooled at a fast, nominal rate of 300 °C/min, held at −60 °C for 5 min and reheated at a rate of 10 °C (=second heating). Thermal analysis was repeated at least three times for every sample. Samples referred to as quench cooled (QC) were obtained by first melting in situ in the DSC and then cooled at a nominal rate of 300 °C/min. Pyris software (version 9.01.0174) was used to analyze the thermograms.

### 2.5. Hyperscan DSC (HSDSC)

HSDSC measurements were carried out using a PerkinElmer Diamond DSC unit described in Section 2.4 using helium at a flow rate of 60 mL/min as the purge gas. Approximately 0.5 mg of accurately weighed powder (MT5 Mettler Toledo microbalance) was analyzed (*n* = 3) in 18 µL aluminum pans with covers. Samples were heated from 0 to 110 °C using heating rates of 50, 100 and 300 °C/min.

### 2.6. Powder X-ray Diffraction (PXRD)

Powder X-ray diffraction analysis, in duplicate for every sample studied, was performed with a Rigaku Miniflex II desktop X-ray diffractometer (Tokyo, Japan) set to 30 kV and 15 mA and equipped with a Haskris cooling unit (Elk Grove Village, IL, USA). Ni-filtered Cu Kα radiation (λ = 1.5408 Å) was used. The measurements were carried out in a range of 5–40 2theta degrees at a step size of 0.05° per second at room temperature. The samples were front-loaded onto a low background silicon mount (Rigaku, Tokyo, Japan) [22].

### 2.7. Attenuated Total Reflection Fourier Transform Infrared Spectroscopy (ATR-FTIR)

ATR-FTIR experiments (*n* = 2) were performed using a PerkinElmer Spectrum 100 FTIR spectrometer (Shelton, CT, USA) equipped with a PerkinElmer universal ATR sampling accessory. A spectral range of 650–4000 cm^−1^, resolution of 4 cm^−1^, accumulation of 10 scans and data interval of 1 cm^−1^ were used. Spectrum software version 10.6.0 was used for background correction, normalization, and infrared spectra analysis.

### 2.8. Analysis of Thermal Data

Experimental phase diagrams were constructed using the thermal data obtained as described in Section 2.4. Theoretical phase diagrams of an ideal eutectic mixture, which implies the existence of complete insolubility between the two components at all concentrations, were also calculated. The theoretical phase diagrams were constructed using the Schröder van Laar equation (Equation (1)) [23]:

(1) $$lnx_{i}=-\frac{∆H_{fi}}{R}\left(\frac{1}{T}-\frac{1}{T_{fi}}\right)$$

where *x_i_* is the mole fraction of the component *i* at temperature *T* (in Kelvin), R is the gas constant (R = 8.314 J K^−1^ mol^−1^), Δ*H_fi_* is the molar enthalpy of fusion of component *i*, and *T_fi_* is the melting temperature (onset) of pure component *i* (in Kelvin). Δ*H_fi_* and *T_fi_* were determined by DSC (Section 2.4).

The eutectic composition, i.e., the composition with the maximal Δ*H* of the eutectic peak, was determined for the KET-BEN mixtures using the Tammann plot, which represents Δ*H* of the eutectic peak as a function composition.

Experimental Tgs were compared with those calculated based on the knowledge about the properties of the pure components [24] using the Fox equation (Equation (2)):

(2) $$\frac{1}{Tg_{m}}=\frac{w_{1}}{Tg_{1}}+\frac{w_{2}}{Tg_{2}}$$

where *Tg* is the glass transition temperature (midpoint) (in Kelvin degrees), *w* is the weight fraction concentration in the mixture and the subscripts denote component 1, component 2 and the mixture (m), respectively, [24]. *Tg* of pure components were determined by DSC (Section 2.4).

For the BEN- and TET-containing samples, for which melting was observed in the second heating cycle, the percentage of crystallinity was calculated using the following formula (Equation (3)) [25]:

(3) $$\%\;crystallinity=\frac{∆H}{∆H_{ref}}\cdot 100\%$$

where Δ*H* is the enthalpy of melting obtained from DSC and corrected for the content of either BEN or TET and Δ*H_ref_* is the enthalpy of melting of the reference, which was considered 100% crystalline (i.e., Δ*H* of BEN and TET from the second heating, 132.2 J/g and 116.4 J/g, respectively, as determined experimentally in this work).

### 2.9. Density Functional Theory (DFT) Calculations

Gaussian03 program was employed to optimize the structures of KET, PRO, TET and BEN [26]. The B3LYP/6-311++G(d,p) level of density functional theory (DFT) was used with no constraints on the geometry of molecules imposed. The dipole moment and charges were estimated using the CHelpG algorithm. A number of global electronic descriptors were calculated according to Koopmans’ theorem [27]. The electron affinity (*A*) was expressed in terms of the HOMO (Highest Occupied Molecular Orbital) energy (*E_HOMO_*) and ionization energy (*I*) as the LUMO (Lowest Unoccupied Molecular Orbital) energy (*E_LUMO_*). The following global reactivity descriptors were calculated: energy band gap (Δ*E*), absolute electron negativity (*χ*), absolute hardness (*η*) and electrophilicity index (*ω*) as per the equations published previously [9,28,29]. Wavefunction analysis was also conducted using Multiwfn 3.6 [30,31].

## 3. Results

### 3.1. Chemical Structure and Relevant Physicochemical Properties of Investigated Molecules

**Figure 1:**
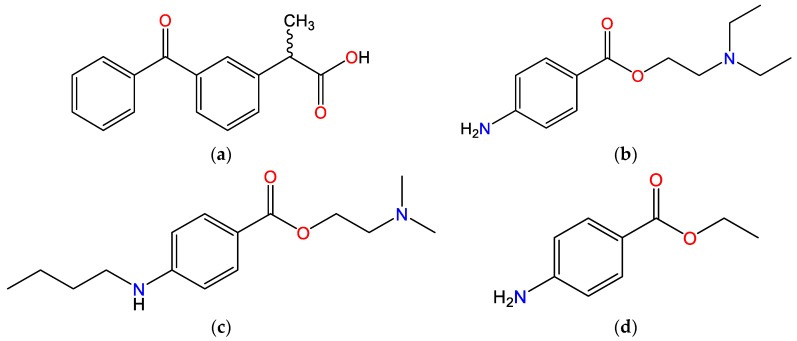
Chemical structures of the investigated molecules: (**a**) ketoprofen (KET), (**b**) procaine (PRO), (**c**) tetracaine (TET) and (**d**) benzocaine (BEN).

The relevant physicochemical properties of investigated molecules are shown in Table 1 and their structural formulas are depicted in Figure 1. KET belongs to the propionic acid class of NSAIDs, it is a weak acid due to the presence of a carboxylic acid group (*pK_a_* = 3.88) and it bears negative charge at physiological pH (7.4). Each molecule of KET has one hydrogen donor and three hydrogen acceptors. TET (also known as amethocaine), PRO and BEN are local anesthetic drugs from the amino ester group. The structure of these local anesthetics consists of three components: a lipophilic part, an intermediate aliphatic chain and a hydrophilic (amine) part, with the ester-type linkage between the lipophilic part and the intermediate chain. The p-aminobenzoic group of ester-type LAs is divided into three subgroups based on the structural differences: procaine group, tetracaine group and benzocaine group. Hence, the investigated molecules are key representatives of each of these groups. PRO has the unsubstituted p-aminobenzoic group and tertiary amine as the hydrophilic group. TET also has the tertiary amine group as the hydrophilic group, but in contrast to PRO, it has a substituted p-aminobenzoic group. BEN is an ester of p-aminobenzoic acid with an unsubstituted amino group and it lacks the terminal tertiary amine group. Except for BEN, the *pK_a_* of the tested LAs is greater than 8, so they are positively charged at pH 7.4. All LAs tested contain one hydrogen donor per molecule. TET and PRO contain three hydrogen acceptors per molecule, whereas BEN contains two hydrogen acceptors.

### 3.2. Ketoprofen-Procaine (KET-PRO) Systems

**Figure 2:**
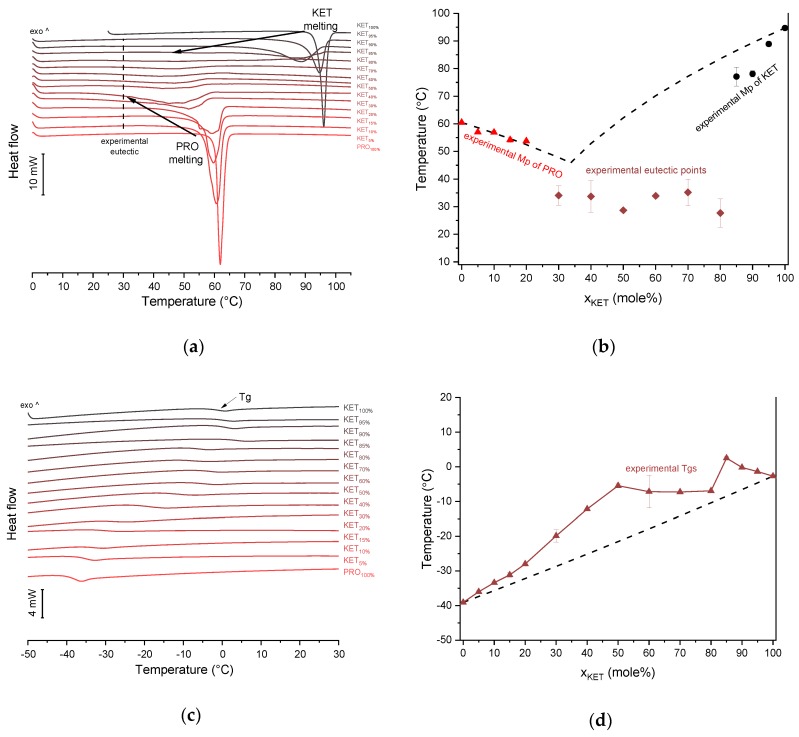
Thermal analysis of KET-PRO systems: (**a**) DSC thermograms from first heating; the broken line indicates the position of the eutectic peak while the arrows show the position of the melting peaks, (**b**) phase diagram based on thermal analysis of first DSC heating; the broken lines show the theoretical liquidus curves calculated using Equation (1), (**c**) DSC thermograms from second heating (the samples were first heated to 110 °C at 10 °C/min, quench cooled at a nominal cooling rate of 300 °C/min and reheated at 10 °C/min) (**d**) phase diagram based on thermal analysis of second DSC heating; the broken line shows the theoretical Tg values calculated using Equation (2). For plots (a) and (c) the subscript indicates the content of the named component in mole%.

KET starting material in the first heating cycle showed a single melting endotherm with an onset at 94.7 ± 0.4 °C. PRO starting material in the first heating also exhibited a single melting endotherm (onset: 60.5 ± 0.1 °C). The heat treatment of the binary KET-PRO mixtures resulted in the formation of a eutectic phase (Figure 2a) and a phase transition to viscous liquids. The Mps of KET and PRO decreased, and the melting endotherms became broader corresponding to their decreasing content in the mixtures. DSC traces of the KET-PRO samples containing 30–80 mol% of KET showed a very broad endotherm with an onset at around 30 °C, which had a low enthalpy of transition. This event can be considered as melting of the eutectic phase. The experimental Mps of PRO in samples containing 5–20 mol% of KET agreed with the predicted values calculated using the Schröder van Laar equation Equation (1), whereas the Mps of KET were lower (Figure 2b). The experimental eutectic temperature was also lower than that determined from Equation (1), which was 46 °C. Therefore, the behavior of PRO and KET in KET-rich mixtures cannot be considered as that of an ideal system, where no strong intermolecular interactions take place. The calculated eutectic composition is 33 mol% of KET, but it was impossible to determine the experimental eutectic composition, due to an overlapping peak of PRO melting.

**Figure 3:**
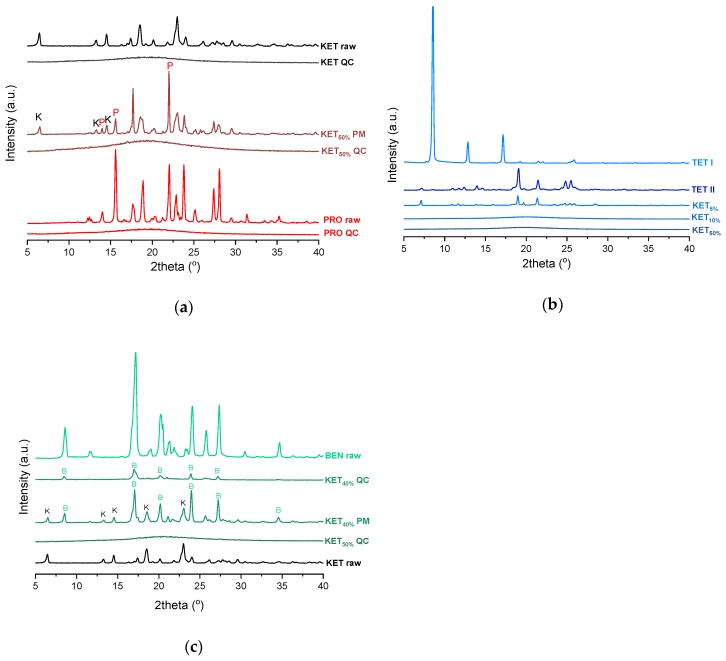
PXRD of: (**a**) KET, PRO and equimolar KET-PRO mixtures; (**b**) KET, TET and KET-TET mixtures; (**c**) KET, BEN and KET-BEN mixtures. QC-quench cooled, PM-physical mixture, raw-as supplied, TET I-TET polymorphic form I, TET II-TET polymorphic form II. The subscript indicates the KET content in mole%.

**Figure 4:**
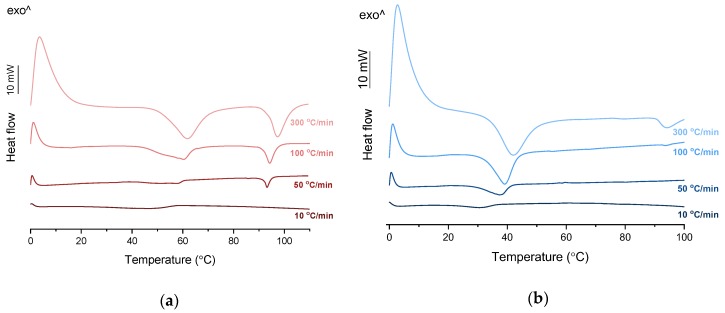
HSDSC thermograms of equimolar KET-LA powder mixtures heated at different rates: (**a**) KET-PRO and (**b**) KET-TET.

PXRD analysis of the KET-PRO physical mixtures prior to the heat treatment revealed the presence of crystalline substances with the peaks corresponding to both, PRO and KET starting materials (Figure 3a). The phase behavior of an equimolar KET-PRO mixture was further investigated by HSDSC (Figure 4a). When heated at a standard rate (10 °C/min), a very broad endotherm with an onset at 28.7 ± 0.4 °C was observed, which can be attributed to the eutectic melting followed by melting/dissolution of the excess of either KET or PRO. The onset of this eutectic endotherm moved from 40.8 ± 1.6 °C to 50.6 ± 1.5 °C when the heating rate increased from 50 °C/min to 300 °C/min and the peak became narrower. Moreover, in the equimolar mixture, at heating rates 50–300 °C/min, a second endotherm with an onset at approximately 92 °C appeared, which was attributed to KET. KET and PRO starting materials at heating rates 50–300 °C/min showed a single narrow melting endotherm with an onset at approximately 92 and 57 °C, respectively, independent on the heating rate (data not shown). Thus, the fast thermal treatment prevented the eutectic phase formation and a phase separation occurred, indicating that the eutectic was, indeed, composed of KET and PRO.

KET did not crystallize either during the fast cooling step or during the second heating (10 °C/min) as evidenced by the lack of the endothermic events and by a glass transition event (*Tg*) in the second heating cycle (midpoint: −2.7 ± 0.5 °C) (Figure 2c,d). Similarly, PRO did not crystallize either during cooling or during the second heating cycle and exhibited a *Tg* at −39.1 ± 0.4 °C. Quench cooled (QC) PRO and KET were disordered by PXRD (Figure 3a). In the phase diagram of the heat-treated KET-PRO samples (Figure 2c) only one phase transition was observed, i.e., the *Tg*, consistent with the disordered “halo” pattern recorded by PXRD (Figure 3a). The presence of one *Tg* in the binary mixtures implies mixing of the components at the molecular level. The *Tg* values, which were dependent on mixture composition (Figure 2d), indicate that heat-treated KET-PRO mixtures formed supercooled liquids. The experimental *Tg* values of samples containing at least 30 mol% of KET were larger than the predicted values calculated by the Fox equation (Equation (2)), thereby implying strong interactions between PRO and KET in the glassy state, which could involve proton transfer or at least strong H-bonds. KET-PRO supercooled liquids showed no tendency to crystallize when heated at 10 °C/min supported by the lack of crystallization exotherms and melting endotherms during the second heating. Moreover, slower heating and/or cooling rates (2 °C/min) did not induce crystallization.

**Figure 5:**
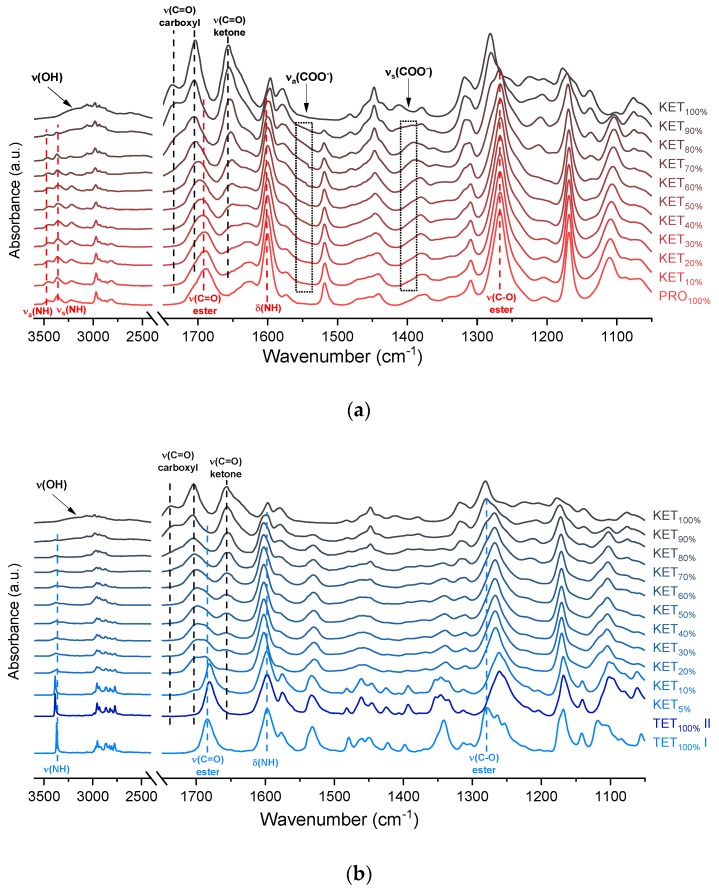

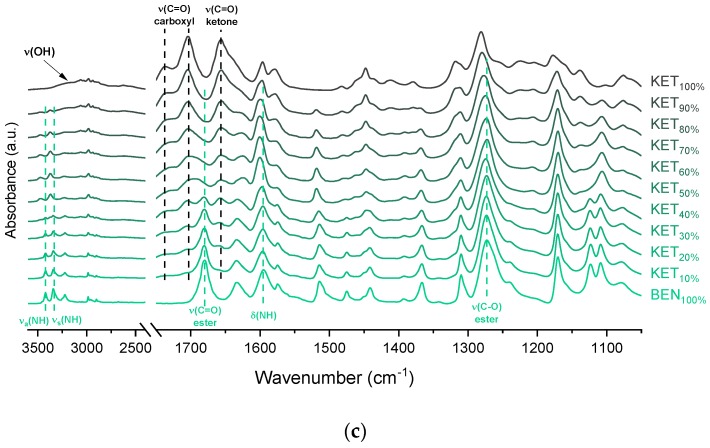
Infrared spectra of: (**a**) quench-cooled KET, PRO and KET-PRO mixtures; (**b**) quench-cooled KET, TET I, TET II and KET-PRO mixtures and (**c**) quench-cooled KET, BEN and KET-BEN mixtures. *ν*—stretching, *ν_a_*—asymmetric stretching, *ν_s_*—symmetric stretching and Δ—bending vibrations. The subscript indicates the content of the named component in mole%.

The infrared spectra of QC PRO, KET and KET-PRO mixtures are shown in Figure 5a. The band at 1695 cm^−1^ in the crystalline KET (starting material) can be ascribed to the C=O stretching of the carboxylic groups of KET molecules organized in dimers in the crystal lattice [33]. In the QC KET, which contained carboxylic acid monomers due to release of KET molecules from crystal lattice, this band shifted towards higher wavenumbers, giving a peak with the center at 1704 cm^−1^ and shoulder at 1737 cm^−1^. The band at 1655 cm^−1^, assigned to the stretching mode of the ketone group [33], was observed at 1656 cm^−1^ in QC KET. The broad peak in the range 2200–3400 cm^−1^ observed in both, KET starting material and QC KET can be assigned to the O–H stretching [33]. It overlaps with the alkane C–H stretching band at 2800–3000 cm^−1^ [34]. In the spectrum of crystalline PRO, two sharp peaks at 3463 and 3366 cm^−1^ can be ascribed to the N–H stretching bands [35]. The NH_2_ groups in the crystal structure form intermolecular hydrogen bond with the ester C=O group [36]. The intensity of these N–H stretching bands decreased drastically in the QC PRO. The band at 1600 cm^−1^ can be attributed to the NH_2_ scissoring mode [35]. The band at 1665 cm^−1^ in PRO starting material is probably the ester C=O stretching [35]. In the spectrum of QC PRO this band shifted towards higher wavenumbers (1689 cm^−1^). The band at 1272 cm^−1^, the most intensive band in the spectrum of PRO starting material, can be attributed to the ester C–O stretching [35]. The band at 1272 cm^−1^, most likely of the C–O stretching, shifted towards lower wavenumbers (1267 cm^−1^) in the spectrum of heated PRO. In the spectra of the QC KET-PRO mixtures, the doublet of N–H stretching bands shifted towards lower wavenumbers (3444 and 3361 cm^−1^ for equimolar mixture), but in the samples containing 70–90 mol% of KET it shifted back towards higher wavenumbers. The broad O–H stretching band was present in the sample containing 90 mol% of KET, but it was difficult to observe it in the sample containing 80 mol% of KET. The position of the N–H scissoring band (1600 cm^−1^) did not change in QC KET-PRO mixtures, neither did the position of the ester C–O stretching band. The ketone C=O stretching band shifted slightly towards lower wavenumbers (1652 cm^−1^ in the equimolar mixture), and in the sample containing 10 mol% of KET it was no longer observed. In the case of the ester C=O band of PRO (1689 cm^−1^) and carboxyl C=O band of KET (1704 cm^−1^), the gradual change of one band into another was observed, in the equimolar sample the position of the ‘hybrid’ band was 1697 cm^−1^. The shoulder of the KET carboxyl C=O monomers shifted towards lower wavenumbers in samples containing 90–70 mol% of KET and it was no longer observed in the sample containing 60 mol% of KET. Hence, the carboxyl group of KET is strongly involved in interactions with PRO molecules. The carboxylate group bands should appear at 1650–1550 cm^−1^ and 1400 cm^−1^, arising from asymmetric and symmetric stretching, respectively, with the former that should be intensive and the latter weak. An increased absorbance in the region above 1550 cm^−1^ was observed in the spectra of QC KET-PRO samples. It was the most intensive in samples containing 60–70 mol% of KET. Hence, it is possible that a fraction of KET molecules is ionized in the supercooled mixtures and the proton is transferred probably to tertiary amine of PRO.

### 3.3. Ketoprofen-Tetracaine (KET-TET) Systems

**Figure 6:**
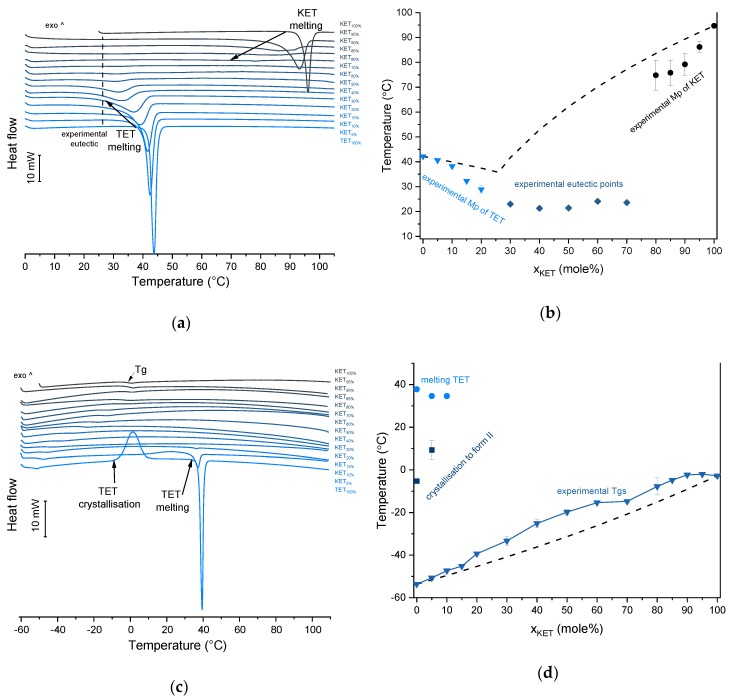
Thermal analysis of KET-TET systems: (**a**) DSC thermograms from first heating; the broken line indicates the position of the eutectic peak while the arrows show the position of the melting peaks, (**b**) phase diagram based on thermal analysis of first DSC heating; the broken lines show the theoretical liquidus curves calculated using Equation (1), (**c**) DSC thermograms from second heating (the samples were first heated 110 °C at 10 °C/min, quench cooled at a nominal cooling rate of 300 °C/min and reheated at 10 °C/min) (**d**) phase diagram based on thermal analysis of second DSC heating; the broken line shows the theoretical Tg values calculated using Equation (2). For plots (a) and (c) the subscript indicates the content of the named component in mole%.

TET showed a single melting endotherm (onset: 42.2 ± 0.0 °C) in the first heating (Figure 6a,b). The Mp of KET and TET decreased and the melting endotherms became broader corresponding to the decreasing quantity of the drugs in the sample. The KET-TET samples with 5–10 mol% of KET showed an Mp similar to that calculated using the Schröder van Laar equation, whereas all other experimental Mps were lower than those predicted for ideal systems. Similar to the KET-PRO mixtures, the formation of a eutectic phase was observed. A decrease in Mp in mixtures containing 15–20 mol% of KET may be explained by accelerated dissolution of TET crystals in the presence of the liquid phase. The eutectic point is probably close to that observed for 30–70 mol% of KET mixtures with an onset of approximately 21–23 °C, hence it is lower than the value calculated for an ideal system (35.8 °C). The theoretical eutectic composition determined from Equation (1) was 26 mol% of KET, but it was impossible to determine the experimental composition, due to overlapping peak of the TET melting endotherm (Figure 6a). PXRD revealed that the equimolar sample (unheated) displayed a pattern corresponding to the mixture of crystalline TET and KET (Figure 3b). To better understand the phase behavior of KET-TET systems, the equimolar physical mixture was heated at different rates. At 300 and 100 °C/min two melting endotherms were observed; the first one that appeared at approximately 33.7 ± 0.9 °C, and the second one had an onset above 90 °C (melting of KET) (Figure 4b). When the heating rate was reduced to 50 °C/min, the first endotherm appeared at lower temperature (30.6 ± 1.3 °C) and was broader, and the melting endotherm of KET was no longer observed. Upon heating at 50–300 °C/min TET showed a sharp melting event at 38 °C (data not shown). Hence, the eutectic and either KET or TET were present in the sample, but at a standard heating rate of 10 °C/min dissolution of KET and/or TET crystals dispersed in the liquid phase can take place resulting in a broad and flat endotherm.

After rapid cooling, TET formed a supercooled liquid with a glass transition at −53.6 ± 0.1 °C (Figure 6c,d). Upon second heating, supercooled TET showed a crystallization exotherm (onset: −5.2 ± 0.3 °C, Δ*H* = 102.8 ± 2.7 J/g) followed by a melting endotherm (onset: 37.8 ± 0.1 °C, Δ*H* = 116.4 ± 0.7 J/g). Since the value of crystallization Δ*H* is approximately 88% of melting Δ*H*, the crystallization occurs mainly during re-heating, but it is possible that the sample partially crystallized during the cooling step. The melting of TET in the second cycle occurred at a lower temperature compared with that from the first heating cycle (onsets 37.8 ± 0.2 °C and 42.2 ± 0.1 °C, respectively). Moreover, the PXRD pattern of QC TET that was heated past the crystallization in the DSC was different than that of the starting material (Figure 3b), indicating a different polymorphic form. Two polymorphs, II and I, have been reported for TET with melting points at 37 °C and 42 °C, respectively [37]. Hence, the starting material is most likely polymorph I (TET I), whereas the product obtained by crystallization during the second heating (heating of supercooled TET) is form II (TET II). The sample containing 5 mol% of KET formed a supercooled liquid and upon heating only 15% of TET crystallized into TET II, the rest of TET remained in the supercooled state. The TET crystallization peak for this sample appeared at higher temperatures (onset: 9.3 ± 4.4 °C) and was broader compared with that of 100% TET. The enthalpy of crystallization was the same as the enthalpy of melting, hence the crystallization process took place during heating. The QC sample containing 10 mol% of KET was disordered by PXRD, but a small quantity of crystalline TET (less than 1%) was detected by DSC. All other QC KET-TET mixtures (15–95 mol% of KET) exhibited only one phase change, a *Tg*, indicating mixing TET and KET at molecular level. The *Tg* values were dependent on the composition and they increased corresponding to an increasing quantity of KET, but in the KET-rich samples (90 and 95 mol% of KET) the Tgs reached a maximum and this temperature was the same as that of 100% KET. The Tgs of the QC mixtures samples containing at least 20 mol% of KET were higher than those calculated from Fox equation (Figure 6d), thereby implying important deviations from the behavior of an ideal system due to interactions between KET and TET.

The infrared spectrum of TET starting material (TET I) showed a single sharp band at 3371 cm^−1^ ascribed to the N–H stretching of the secondary aromatic amino group (Figure 5b), which shifted towards a higher wavenumber in TET II (3387 cm^−1^). In the spectrum of TET I the stretching of C=O and C–O ester bonds produced bands at 1684 cm^−1^ and 1279 cm^−1^, respectively, [35,38] and they shifted to lower wavenumbers (1681 cm^−1^ and 1261 cm^−1^, respectively) in TET II. The presence of TET II was also observed in the spectrum of the mixture containing 5 mol% of KET. In the sample comprising 10 mol% of KET broadening and a dramatic decrease in the intensity of the N–H stretching band (3375 cm^−1^) was observed due to TET molecules being able to interact with KET. The O–H stretching band was detected in the sample containing 90 mol% of KET, and it was difficult to observe it in the samples containing a smaller amount of KET. The position of the ester stretching C–O band was depended on the solid-state properties of the sample: it shifted from 1279 cm^−1^ (TET I) to 1261 cm^−1^ in TET II, and in the QC KET-TET samples it was present at 1267 cm^−1^. A decreased intensity of the ester C=O stretching band was observed in the QC samples. In the sample containing 10 mol% of KET it was localized at 1688 cm^−1^ and it shifted towards a higher wavenumber corresponding to an increasing KET concentration and overlapped with the carboxyl C=O stretching band. The latter band shifted towards a lower wavenumber and its intensity decreased corresponding to a decreasing KET concentration. The shoulder of the carboxyl C=O stretching band localized at 1737 cm^−1^ (100% KET) also shifted towards lower wavenumbers and was no longer observed in the sample containing 60 mol% of KET, similar to the KET-PRO system. The IR results indicate that TET interact with KET by strong hydrogen bonding, and the carboxyl group of KET is involved in the interactions. Interactions between KET and TET are sufficiently strong to break the crystal lattice of TET even at KET concentrations as low as 10 mol%. However, in contrast to KET-PRO, in the KET-TET samples carboxylate anion was not detected.

### 3.4. Ketoprofen-Benzocaine (KET-BEN) Systems

**Figure 7:**
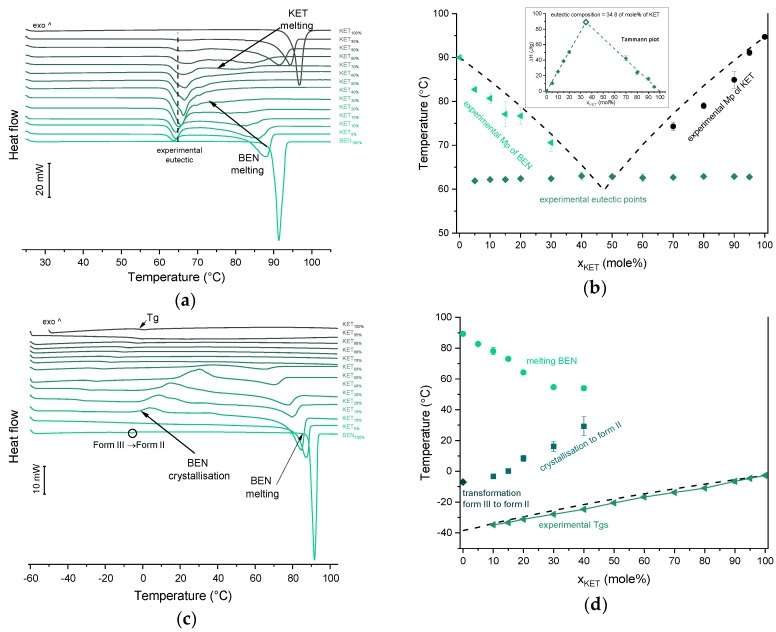
Thermal analysis of KET-BEN systems: (**a**) DSC thermograms from first heating; the broken line indicates the position of the eutectic peak while the arrows show the position of the melting peaks, (**b**) phase diagram based on thermal analysis of first DSC heating; the broken lines show the theoretical liquidus curves calculated using Equation (1). The Tammann plot is shown in the inset; the broken lines present the best linear fits to the experimental data, (**c**) DSC thermograms from second heating (the samples were first heated 110 °C at 10 °C/min, quench cooled at a nominal cooling rate of 300 °C/min and reheated at 10 °C/min) (**d**) phase diagram based on thermal analysis of second DSC heating; the broken line shows the theoretical Tg values calculated using Equation (2). For plots (a) and (c) the subscript indicates the content of the named component in mole%.

BEN exhibits polymorphism and three polymorphic forms have been described to date. Form I, formerly known as form β, is a monoclinic P 2_1_/c polymorph (Z = 4) [39,40]. Form II, formerly known as α, is orthorhombic with space group P2_1_2_1_2_1_ (Z = 4) [40,41]. Form III is another monoclinic P2_1_ polymorph (Z = 8) [40,42]. Polymorph II (referred to as Mod I^0^ by Schmidt, [43]) is thermodynamically stable under ambient conditions, and is present in commercial products. The starting material used in our study was form II. In the first heating cycle BEN showed a single melting endotherm (onset: 90.0 ± 0.1 °C). The KET-BEN mixtures formed a eutectic phase (Figure 7a). Both, KET and BEN melted at lower temperatures and had broader melting endotherms corresponding to their decreasing content in the mixture. Experimental Mps of KET and BEN showed an agreement with values calculated using the Schröder van Laar equation. All binary KET-BEN mixtures showed eutectic peaks with an onset at approximately 62–63 °C. The shape of the eutectic peak depended on the composition: the peaks were broader in the KET-rich samples than in the BEN-rich samples. It was difficult to discern individual peaks in the mixtures containing 30–70 mol% of KET. Thus, to construct the Tammann plot (Δ*H* of the eutectic peak as a function composition), only the mixtures containing 5–20 and 80–95 mol% of KET were taken. The eutectic composition determined using the Tammann plot was 34.8 of mol% of KET (Figure 7b). This composition contains less KET that the theoretical eutectic composition determined by Equation (1), which is 47 mol% KET. Assuming that this eutectic phase was a simple mechanical mix, the enthalpy of mixing (Δ*H_mix_*) was calculated using Equation (4) [23]:

(4) $$∆H_{mix}={\left(∆H_{fus}\right)}_{exp}-\left(x_{\mathrm{KET}}\cdot ∆H_{fus\mathrm{KET}}+x_{\mathrm{BEN}}\cdot ∆H_{fus\mathrm{BEN}}\right)$$

where (Δ*H_fus_*)*_exp_* is the heat of fusion of the eutectic peak at the eutectic composition obtained from the Tammann plot, *x* is the mole fraction of the component (KET or BEN) and Δ*H_fus_* is the heat of fusion of the pure constituent (KET or BEN). The value of Δ*H_mix_* for the KET-BEN system was negative, –2.33 kJ/mol, indicating the presence of weak intermolecular forces leading to cluster formation [44]. The KET-BEN physical mixtures showed peaks characteristic of starting materials in X-ray diffractograms (Figure 3c).

In the second heating cycle BEN melting endotherm was observed at the same temperature as in the first heating, but it was preceded by a small endotherm at −7.0 ± 0.1 °C (Δ*H* = 1.0 ± 0.2 J/g) (Figure 7c,d). Interestingly, Gana et al., [45] observed a similar endothermic event, which was attributed to a phase transition of form III into form II. Forms II and III are enantiotropic under ambient conditions. Form III is stable at low temperature and form II is stable at higher temperature and melts eventually. Form III becomes more stable with increasing pressure at ambient temperature [45]. Form II was detected in the QC BEN by PXRD (Figure 3c). After a rapid cooling step of the KET-BEN melt, a supercooled liquid was formed, except for the mixture containing 5 mol% of KET. A *Tg* was detectable down to the KET content of 10 mol%. A very strong linear relationship was observed between the composition (mol% of either BEN or KET) and the *Tg* (*R^2^* = 0.999). It was not achievable to obtain BEN 100% in the supercooled state, because it crystallized during cooling despite the very fast cooling rate employed. Hence, to determine the calculated Tgs using the Fox equation, the *Tg* of BEN 100% (−38.5 °C) was determined by extrapolation. The experimental Tgs showed a good agreement with the calculated Tgs. This implies the behavior of an ideal mixture, in which the tendency of two kinds of molecules (BEN and KET) to transfer from the glassy state to the supercooled liquid state is unchanged. The presence of only one *Tg* in the binary mixtures containing at least 10 mol% of KET indicates that KET and BEN are miscible. The amount of crystalline of BEN decreased from 86.0 ± 0.7% (5 mol% of KET) to 15.2 ± 7.8% (40 mol% of KET), and crystalline BEN was no longer observed in samples containing at least 50 mol% of KET. QC mixtures that contained at least 50 mol% of KET showed a halo-pattern characteristic of disordered materials (Figure 3c). Hence, the increasing concentration of KET increased the amount of BEN entrapped in the supercooled state. In the sample containing 5 mol% of KET, crystallization of BEN took place during cooling. The crystallization of BEN was viewed during the second heating for mixtures containing 40–90 mol% of KET. The onset temperature of the crystallization peak increased corresponding to a decreasing BEN concentration in the sample. The percentage of BEN that crystallized during heating increased from 23% (10 mol% of KET) to approximately 100% in mixtures containing 20–40 mol% of KET. X-ray diffractograms confirmed that it is BEN that crystallizes, because only BEN peaks were observed in the X-ray diffractograms of heated, previously QC KET-BEN systems (KET ≤ 40 mol%) (Figure 3c). Some samples, such as that containing 10 mol% of KET, showed a multistep crystallization process, which could be the interfacial crystallization (first part of the peak) that was followed by bulk crystallization (second part of the peak). PXRD patterns of this QC sample that was heated in the second heating cycle to either 20 °C or 50 °C were the same and showed peaks characteristic for BEN (data not shown). The BEN crystallization peak was followed by the melting peak with an onset temperature decreasing corresponding to a decreasing BEN content, from 78.1 ± 2.3 °C to 54.0 ± 1.6 °C for KET 10 and 40 mol%, respectively. Δ*H* values of the BEN melting peak in the second heating cycle were markedly higher than those observed in the first heating cycle for the mixtures containing 5–30 mol% of KET.

The interactions between BEN and KET were further examined by ATR-FTIR (Figure 5c). The doublet consisting of two bands at 3421 and 3340 cm^−1^ can be ascribed to asymmetric and symmetric N–H stretching bands, respectively [35,40]. The scissoring band of NH_2_ is present at 1595 cm^−1^. The C=O and C–O bonds of aromatic esters produce bands at 1680 and 1273 cm^−1^, respectively [35]. The latter may overlap with the C–N stretching band characteristic of aromatic amines in the range 1240–1366 cm^−1^ [35]. In the spectra of QC KET-BEN mixtures the positions of bands at 1273 cm^−1^ of the ester C-O stretching and at 1656 cm^−1^ of the stretching of ketone C=O group did not change markedly. In mixtures containing at least 50 mol% of KET the bands at 3421 and 3340 cm^−1^ of the asymmetric and symmetric N–H stretching bands disappeared, and new bands at 3472 and 3371 cm^−1^ were observed. The latter bands became noticeable in samples containing 20–30 mol% of KET. Interestingly, in the sample comprising KET 40 mol% both, the two former (3421 and 3340 cm^−1^) and the two latter (3472 and 3371 cm^−1^) bands were easily observed. The presence of those bands is in a good agreement with the crystallinity of BEN determined by PXRD and DSC. The packing of BEN molecules in crystals is stabilized by N–H···O hydrogen bonds [42]. Hence, the bands at 3421 and 3340 cm^−1^ correspond to N–H stretches of the NH_2_ group in the crystal lattice that interact via H-bonding with the C=O moiety of the ester groups, and the increase in wavenumber to 3472 and 3371 cm^−1^ is probably due to the liberation of the NH_2_ groups from the crystal lattice due to interactions with KET. This is consistent with the fact that the band of the ester C=O stretching of BEN (1680 cm^−1^) was no longer detectable in the equimolar KET-BEN mixture. The peaks characteristic of the NH_2_ group in the crystal lattice disappear in the samples that do not contain a detectable amount of crystalline BEN (at least 50 mol% of KET). The intensity of the bands characteristic of ‘supercooled’ NH_2_ groups also agree well with the content of noncrystalline BEN in the sample. The band of the NH scissoring at 1595 cm^−1^ shifted towards higher wavenumbers (1600 cm^−1^) in the QC samples containing 50–80 mol% of KET, the band shifted to 1597 cm^−1^ for the sample containing 90 mol% of KET because it was dominated by the C–C stretching band of KET at 1596 cm^−1^ [34]. The intensity of the broad O–H stretching band decreased corresponding to a decreasing KET concentration. Interestingly, this band was observed in the sample containing 60 mol% of KET, whereas in the PRO and TET mixtures it was only observed at KET 90 mol%. Also, both shoulders of the C=O stretching group band of the carboxyl acid monomer of KET (1704 and 1737 cm^−1^) moved to lower wavenumber corresponding to a decreasing KET concentration (1693 and 1727 cm^−1^ for the equimolar KET-BEN mixture). It can be concluded that KET interacts BEN via H-bonding, as suggested by the negative value of Δ*H_mix_*, thereby decreasing the interactions between BEN molecules in the crystal lattice and leading to entrapment of BEN in the supercooled state.

### 3.5. DFT Studies

Molecular modelling studies were carried out to determine the likelihood and mode of intermolecular interactions in the binary systems. The global reactivity parameters are listed in Table 2. For LAs, the higher HOMO energy was calculated for TET, therefore this molecule should be the best electron donor, while the lowest LUMO energy was determined for BEN and PRO, implying the best electron acceptor properties of these two substances [46]. The values of I and A also suggested the same characteristics. Electronegativity values indicate if a molecule is a Lewis acid (large *χ*) or a base (low *χ*), therefore it can be said that KET will act as a Lewis acid (also consistent with the high value of *ω*) [47]. The molecule that is deemed as most reactive, based on the chemical hardness is TET followed closely by PRO. The global reactivity parameters, however, do not show a similar trend as that observed from the experimental studies, where PRO interacted with KET most strongly, followed by TET and BEN. It is because the calculated values do not include any effects of ionization (proton transfer), that possibly occur between KET and PRO.

**Figure 8:**
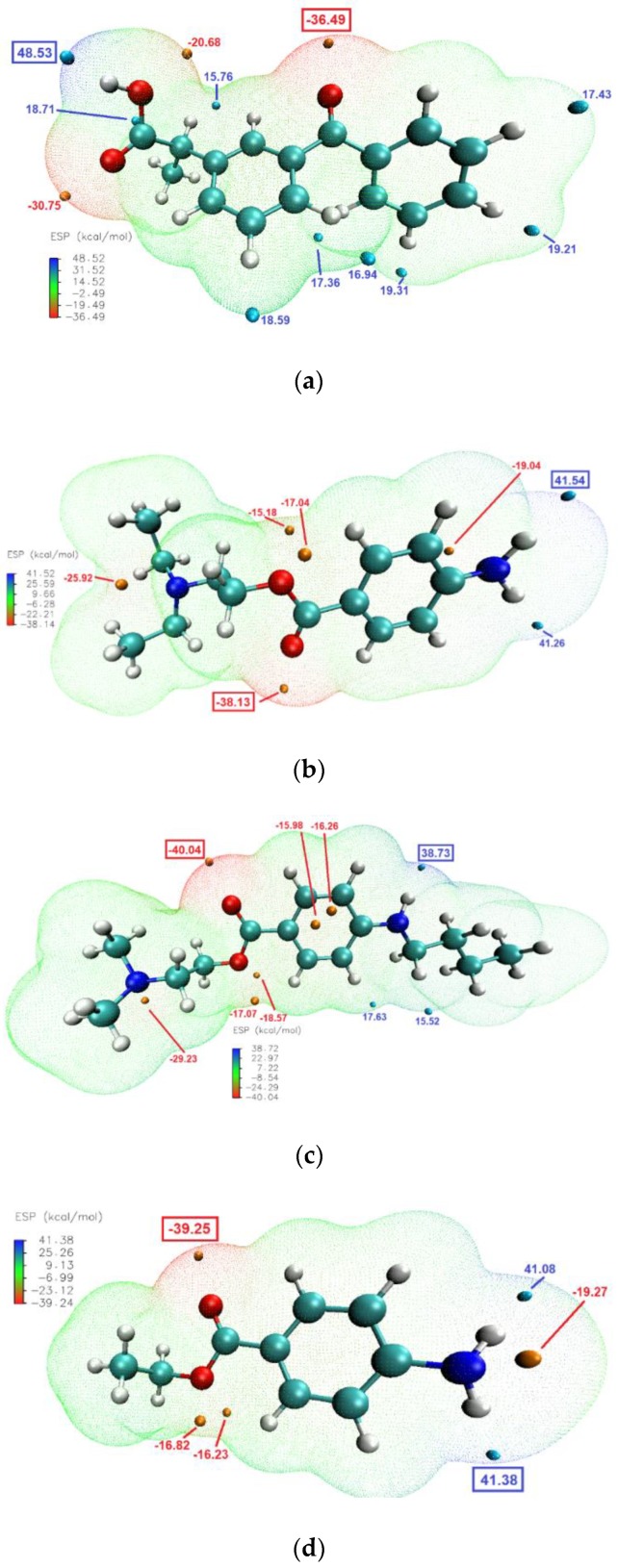
Electrostatic potential of: (**a**) KET, (**b**) PRO, (**c**) TET and (**d**) BEN mapped on the 0.001 au contours of the electron density of the molecules optimized by DFT. The negative regions are indicated in blue, while the positive regions are shown in red. Surface local minima and maxima (only values > 15 kcal/mol) are represented as orange and cyan points, respectively. The global maximum and minimum for every molecule are shown in boxes in large font. The unit is kcal/mol.

The molecular electrostatic potential (ESP) calculations can be vital in predicting intermolecular interactions [48]. Mapping the ESP on molecular vdW surface of KET and the studied LAs (Figure 8), clearly showed that the most likely intermolecular interactions between KET and each of the LAs will be between the –OH moiety of the carboxylic group of KET (ESP maximum of 48.5 kcal/mol) and the carbonyl of the ester moiety of the LA (ESP minimum of −38.13, −40.04 and −39.25 kcal/mol for PRO, TET and BEN, respectively). This is consistent with the results of the infrared analysis presented above, evidencing H-bond formation in the binary systems.

### 3.6. General Discussion

Multicomponent mixtures were successfully produced by the mechanosynthetic process, which involved mixing of an acid (KET) with a base (LA) in a range of molar ratios followed by heating. This process has important advantages. It does not necessitate the use of solvent, hence there is no need for product drying and determining the residual solvent content. Another advantage is the high product yield and purity because the process does not require product isolation and/or purification. Thus, the mechanosynthesis eliminates the drawbacks of commonly used metathesis reactions. The mechanosynthetic process described in this paper is applicable for NSAID-LA combinations and other DEMs/ILs. If the chosen combination contains substances that decompose upon melting (such as acetylsalicylic acid or diclofenac), solvent assisted grinding such as that described previously [20] for ethambutol ILs/salts or aspirin cocrystals [49] could be an alternative.

The heat treatment of a binary KET-LA physical mixtures resulted in the formation of eutectic phase. The thermograms of the binary KET-TET and KET-PRO mixtures contained broad endotherms with low intensity, except of mixtures containing very large excess of one of the components. It may be due to the fact that a part of dispersed crystals is surrounded by the liquid phase generated by the melting of the eutectic. This can induce the accelerated melting and/or dissolution of the component in excess (either KET or LA). Kataoka et al., [14] observed a similar phenomenon for ibuprofen-lidocaine binary mixture. The formation of eutectic mixtures has been observed for another NSAID–LA pair: indomethacin-lidocaine [18]. The eutectic peak in the KET-LA systems studied very often overlapped with the subsequent melting events. KET-PRO and KET-TET, except of LA-rich mixtures, showed important deviations from the behavior of an ideal system due to interactions between the KET and LA molecules, hence it was impossible to determine the eutectic composition. The behavior of the KET-BEN system, on the other hand, agreed with the theoretical predictions. The eutectic peaks were better separated from the succeeding thermal events compared with KET-TET and KET-PRO, and it was possible to determine the KET-BEN eutectic composition using the Tammann plot.

In the phase diagrams from the second heating, all KET-PRO samples were characterized by a single-phase transition from glass to the supercooled state. Similarly, only one *Tg* was observed in the samples containing 5–85 mol% of TET and 5–50 mol% of BEN. The presence of only one *Tg* confirms that KET and LAs are mixed at molecular level. KET-BEN and LA-rich KET-TET and KET-PRO mixtures showed a good agreement with theoretical predictions of *Tg* by Fox equation, however in the other KET-TET and KET-PRO combinations a considerable increase in *Tg* compared with predicted value was observed. This may imply the existence of strong KET-TET and KET-PRO interactions in the glassy state. During the second heating in the mixture containing 95 mol% of BEN only one melting was observed, whereas in the samples containing 60–90% of BEN and 95 mol% of TET three phase transitions were observed: a *Tg*, followed by LA crystallization and LA melting. The crystallization was always to the pure LA (either TET or BEN). Active pharmaceutical ingredients can be categorized into three different classes based on their crystallization tendency [16]. Despite the similarity of their structures, the tested LAs show a wide variation in crystallization tendency. BEN has been categorized as class I molecule, because it crystallizes during cooling from the undercooled melt state prior to the *Tg* event [16]. Hence, it has high crystallization tendency and low glass-forming ability. On the other hand, PRO and KET are class III molecules, with low crystallization tendency and high glass-forming ability for which no crystallization occurs upon either cooling to below *Tg* or upon subsequent reheating up to the melting point [16]. Baird et al. [16] did not investigate TET, but based on our results it can be categorized as a class II molecule, for which no crystallization is observed upon cooling from the undercooled melt state to below *Tg*, however, crystallization is observed during reheating above *Tg*. Although BEN has a very low glass-forming ability, after mixing with KET it was possible to capture some BEN molecules in the supercooled state. Interactions between KET and TET are sufficiently strong to break the crystal lattice of TET even at KET concentrations as low as 10 mol%.

The preparation of protic ILs typically demands a sufficient pK_a_ difference between the acid and the base that could lead to an effective proton transfer and formation of an ion pair [6]. The recommended difference of ΔpK_a_ of 10 is not possible to achieve for most APIs [6], including NSAIDs and LAs. The pK_a_ is determined in a diluted aqueous solution, hence its application for tested multicomponent systems in the pure form that are not dissolved/dispersed in water, is limited. The ionization depends not only on the pK_a_, but also on structural features of acid and base [6,8,17]. The DSC and IR results show that interactions between KET and BEN are markedly weaker than those between KET and either TET or PRO. This is consistent with the fact that BEN is weaker base than either of TET or PRO. The interactions between PRO and KET seem to be stronger than those between TET and KET, because there are indications of the presence of a small level of carboxylate anions in the former mixture. Moreover, the positive deviations from the theoretical Tgs that are larger in KET-PRO system (a difference of 16 °C for the equimolar mixture) than in KET-TET system (a difference of 11 °C for mixtures containing 40–60 mol% of KET) imply stronger electrostatic interactions in the former. In systems composed of ciprofloxacin and Eudragit L100, particularly large positive *Tg* deviations (45–48 °C) from theoretical predictions were observed due to ionic interactions [21]. The low degree of proton transfer in the KET-LA systems tested is consistent with the previous observations for similar systems such as ibuprofen-lidocaine [2]. When a tertiary amine is used as the base (PRO and TET in our study) the proton transfer may be severely restricted because of the lack of a satisfactory hydrogen bonding solvation environment for the anionic species formed [2]. In conclusion, all KET-LA systems studies in this work are DEMs, i.e., mixtures of hydrogen bond donors and acceptors with intermolecular interactions via hydrogen bonding.

Although the salt preparation of the APIs with poor solubility in water is one of the most effective and developable approach to improve their solubility and dissolution rate, new salts of the API may be recognized as new chemical entities by the FDA and other healthcare authorities. Therefore, the absence of ion pair formation in the KET-LA systems could be beneficial from the regulatory point of view, as they should not be considered as new chemical entities.

## 4. Conclusions

A mechanosynthetic process that involved mixing and co-melting of a free acid and a free base proved to be applicable for producing KET-LA DEMs. The binary KET-LA powder mixtures formed eutectic phases. After a rapid cooling the formation of a supercooled liquid, with a *Tg* that was highly dependent on the composition, was observed for all mixtures with exception of that containing 95% mol% of BEN. Crystallization of LA was observed for QC samples comprising BEN-rich mixtures (BEN ≥ 60 mol%) and the sample containing TET 95 mol%. In the presence of KET the high crystallization tendency of LAs of TET and BEN can be decreased, and their glass-forming ability increased. The KET-PRO DEMs in the entire composition range did not crystallize either during the cooling step or during the second heating cycle. Experimental Mps and Tgs of KET-BEN mixtures follow the theoretical Schöder van Laar and Fox prediction, respectively. On the other hand, for PRO and TET-based systems, high deviations from the theoretical Schröder van Laar and Fox predictions were observed, indicating the formation of strong H-bonded complexes between these LAs and KET. A small quantity of carboxylate anions was present in the KET-PRO samples, but the proton transfer is severely restricted due to the lack of a satisfactory hydrogen bonding solvation environment necessary for anion stabilization. The strength of interactions with KET can be ranked in the following order: PRO > TET > BEN. Therefore, it can be concluded that KET-LA mixtures do not form ILs, but DEMs.

## Figures and Tables

**Table 1:** Physicochemical properties of the investigated molecules (obtained from [32]).

Molecule	Ketoprofen	Tetracaine	Procaine	Benzocaine
Molecular weight	254.28 g/mol	264.37 g/mol	236.32 g/mol	165.19 g/mol
Melting point ^1^	94.7 ± 0.4 °C	42.2 ± 0.0 °C	60.5 ± 0.1 °C	90.0 ± 0.1 °C
Δ*H* ^1^	106.0 ± 1.3 J/g	144.2 ± 0.2 J/g	106.0 ± 0.5 J/g	129.3 ± 0.7 J/g
pK_a_^2^	3.88	8.42	8.96	2.78
Physiological charge	−1	+1	+1	0
Hydrogen donor counts	1	1	1	1
Hydrogen acceptor counts	3	3	3	2

^1^ Obtained experimentally in this work. ^2^ Strongest acidic for KET, protonated amine for LAs.

**Table 2:** Dipole moments and global reactivity parameters of KET, PRO, TET and BEN. Δ*E*—HOMO/LUMO energy gap, *I*—electron affinity, *A*—ionization potential, *χ*—electronegativity, *η*—hardness and *ω*—electrophilicity index.

	Dipole moment	HOMO (eV)	LUMO (eV)	ΔE (eV)	*I*	*A*	*Χ*	*η*	*ω*
KET	2.4855	−7.048	−2.150	4.898	7.048	2.150	4.599	2.449	4.318
PRO	3.2811	−5.905	−1.143	4.762	5.905	1.143	3.524	2.381	2.608
TET	4.5044	−5.769	−1.034	4.735	5.769	1.034	3.4015	2.3675	2.444
BEN	3.4108	−6.068	−1.143	4.925	6.068	1.143	3.6055	2.4625	2.640
